# Knowledge and Perception of COVID-19 Pandemic during the First Wave (Feb–May 2020): A Cross-Sectional Study among Italian Healthcare Workers

**DOI:** 10.3390/ijerph18073767

**Published:** 2021-04-04

**Authors:** Caterina Rizzo, Ilaria Campagna, Elisabetta Pandolfi, Ileana Croci, Luisa Russo, Sara Ciampini, Francesco Gesualdo, Alberto Eugenio Tozzi, Lara Ricotta, Massimiliano Raponi, Marta Luisa Ciofi Degli Atti

**Affiliations:** 1 Clinical Pathways and Epidemiology, Bambino Gesù Children’s Hospital IRCSS, Viale di Villa Pamphili 100, 00152 Rome, Italy; caterina1.rizzo@opbg.net (C.R.); marta.ciofidegliatti@opbg.net (M.L.C.D.A.); 2 Multifactorial Disease and Complex Disease Research Area, Bambino Gesù Children’s Hospital IRCSS, Viale di Villa Pamphili 100, 00152 Rome, Italy; ilaria.campagna@opbg.net (I.C.); ileana.croci@opbg.net (I.C.); luisa.russo@opbg.net (L.R.); francesco.gesualdo@opbg.net (F.G.); albertoeugenio.tozzi@opbg.net (A.E.T.); 3 Public Health Service, Local Health Authority Rome 1, Borgo Santo Spirito 3, 00193 Rome, Italy; drsara.ciampini@gmail.com; 4 Medical Direction, Bambino Gesù Children’s Hospital IRCCS, Piazza S. Onofrio 4, 00165 Rome, Italy; lara.ricotta@opbg.net (L.R.); massimiliano.raponi@opbg.net (M.R.)

**Keywords:** information, attitude, COVID-19, SARS-CoV-2, healthcare workers

## Abstract

Italy was the first country in Europe to face the coronavirus pandemic. The aim of the study was to analyze healthcare workers’ (HCWs) level of information, practice, and risk perception towards COVID-19. We set up a cross-sectional study through SurveyMonkey^®^ and distributed the link through Facebook and Whatsapp closed groups. The research instrument was a 31 items questionnaire distributed using Facebook and Whatsapp. It was conducted in Italy from February to May 2020. The study participants were general practitioners, pediatricians and other health professionals. A total of 958 participants were included: 320 (33.4%) general practitioners, 248 (25.9%) pediatricians and 390 (40.7%) other health professionals. The highest response rate was from Northern Italy (48.1%), followed by Central Italy (29.9%) and Southern Italy (22.0%). Less than a half (46%) of respondents felt they had a good level of information of COVID-19 case definition and of national prevention guidelines. Respondents reported to have changed their clinical practice; particularly, they increased the use of masks (87.1%, *p* < 0.001), disinfection and sanitization of doctors’ offices (75.8%, *p* < 0.001), the use of protective glasses (71.2%, *p* < 0.001), alcoholic hand solution (71.2%, *p* < 0.001), and hand washing (31.8%, *p* = 0.028). HCWs are at high risk of infection; less than a half of them felt adequately prepared to face COVID-19 pandemic, so they need extensive information and awareness of the disease to take adequate precautionary measures, and they are crucial to disseminate good practices.

## 1. Background

On 31 December, 2019, the World Health Organization (WHO) received reports from the Chinese health authorities about the presence of pneumonia cases of unknown cause detected in the city of Wuhan, in the Chinese province of Hubei [1]. Subsequently, the Chinese health authorities identified a new coronavirus, Severe Acute Respiratory Syndrome–Coronavirus 2 (SARS-CoV-2), as responsible for the coronavirus disease 2019 (COVID-19) [2].

The first two cases of the COVID-19 pandemic in Italy, which tested positive for the SARS-CoV-2 virus in Rome, were confirmed on 30th January, both with a travel history to Wuhan, China. On 21 February, 2020, the Italian National Institute of Health confirmed the first autochthonous case in Northern Italy (Codogno city–Lombardy region) in a critically ill, hospitalized young man with no travel history to known areas of viral circulation or links to a probable or confirmed COVID-19 case [3,4,5].

This unexpected finding unveiled ongoing transmission in several municipalities in the Lombardy Region [6]. In subsequent days and weeks, case counts and death tolls increased rapidly, first in Northern Italy, and then in the rest of the country. The Italian government imposed increasingly strict physical distancing measures, starting with the closure of 10 municipalities in the Lodi Province (Lombardy) and one in the Padua Province (Veneto) on the 22nd of February 2020 [5,6,7]. This culminated in a national lockdown declared on 10 March 2020 and ended on 3 May 2020. COVID-19 is spread by human-to-human transmission through droplets and direct contact; it has an incubation period of 2–14 days [8,9,10,11,12,13].

The clinical presentation of the Sars-CoV-2 infection varies from asymptomatic to very severe pneumonia with acute respiratory distress syndrome, septic shock and multi-organ failure, which can cause death [14,15,16]. To date, applying preventive measures to control COVID-19 infection is the most critical intervention.

Healthcare workers (HCWs) are at high risk of infection and they may also contribute to the spread of the disease. Infection prevention and control (IPC) during health care practices when COVID-19 is suspected or confirmed is crucial in order to protect HCWs and fragile patients [17]. The rate of infection reported in HCWs varies across countries [18,19,20,21]. In China, HCWs accounted for 3.8% of all cases, with 14.8% of these having severe/critical disease despite their young age and few comorbidities [22,23]. Other studies, however, reported lower illness severity in HCWs and identified PPE use as the main factor associated with decreased infection risk [24].

Measuring scope of information, attitudes and risk perception in regards to IPC could help to predict HCWs’ behaviors in applying preventive and control measures.

Even though the Italian Ministry of Health published guidelines and developed strong initiatives for IPC in healthcare facilities to prevent the spread of the disease, it is crucial to understand if those guidelines were really applied, particularly among HCWs [25].

The aim of the study was to the measure information level of healthcare workers, their risk perception towards the pandemic and their practice (behavior change) in applying COVID-19 nonpharmaceutical preventive measures. With this purpose, we conducted a cross-sectional study at the very beginning of the autochthonous circulation of SARS-CoV-2 in Italy.

## 2. Methods

### 2.1. Study Design

This cross-sectional study was conducted in Italy from the end of February to the first week of May 2020, and it was coordinated by Bambino Gesù Children’s Hospital in Rome.

### 2.2. Data Collection Procedures

The survey was set up using Survey Monkey^®^ and the link to the survey was circulated online through Facebook and WhatsApp closed groups. The study participants were HCWs, including general practitioners, pediatricians, consultants, postgraduate trainees, and other health professionals (nurses, midwives, physiotherapists, etc.).

Data was collected using a structured questionnaire that comprised 31 predefined responses, including the demographic, scope of information, awareness and practice assessment sections. The developed questionnaire was tested among different HCWs in the Bambino Gesù Children’s Hospital, and open-ended questions were limited to reduce information bias.

The Section 1 of the questionnaire consisted of six questions regarding demographic details and professional profile. Section 2 consisted of two questions about the risk perception of HCWs and patients. Section 3 consisted of seven questions focusing on the information level of HCWs. Section 4 had 15 questions regarding attitudes and practices, precautions and procedures to contain the virus.

### 2.3. Sample Size

The sample size for the survey was calculated according to the formula adopted in the Raosoft software (http://www.raosoft.com/samplesize.html, accessed on 12 November 2020). Setting the expected proportion of the outcome found in each question of the study at 50% with an accepted margin of error of 5%, we obtained a total sample of 377 individuals, with a confidence level of 95%.

### 2.4. Definitions

We considered two main outcomes in the analysis: information and behavior change. Information was defined as at least an affirmative response to one of the following four questions:

- Do you believe that information released by international health authorities regarding the pandemic from COVID-19 in China has been clear enough?
- Do you believe that information disseminated by national and regional health authorities regarding the risks associated with COVID-19 for the Italian population has been sufficiently clear?
- Do you think that the definition of a suspected case of COVID-19 infection is sufficiently clear?
- Do you think you have been sufficiently informed by the national health authorities on how to behave if you are faced with a suspected case of COVID-19?

Practice (behavior change) was defined as at least an affirmative response to one of the following three questions:

- Since the start of the COVID-19 pandemic, have you changed the way you work?
- Since the start of the COVID-19 pandemic, has there been any impact in the organization of visits?
- Since the beginning of the COVID-19 pandemic, has there been any impact in your relationship with patients?

Questions on the risk perception for being in contact with COVID-19 for HCWs and their patients were measured using the Likert scale ranging from 0 to 10 (no risk and high risk, respectively); the answers were then categorized into five groups according to the percentile distribution to better show the results in the graph.

For the two questions based on the Likert scale, the data were divided in five groups according to a 20% difference.

The first question (“Are the patients you come in contact with, scared of the COVID-19 pandemic?”) was categorized as follows: group 1 (0–20°) was from 0–5 points of the Likert scale; group 2 (21°–40°) was from 6–7 points of the Likert scale; group 3 (41°–60°) corresponded to 8 points of the Likert scale; group 4 (61°–80°) corresponded to 9 points of the Likert scale; group 5 (81°–100°) corresponded to 10 points of the Likert scale.

The second question (“Based on your views, what is the risk of visiting a patient with SARS-CoV-2 in the coming weeks?”) was categorized as follows: group 1 (0–20°) was from 0–5 points of the Likert scale; group 2 (21°–40°) corresponded to 6 points of the Likert scale; group 3 (41°–60°) was from 7–8 points of the Likert scale; group 4 (61°–80°) corresponded to 9 points of the Likert scale; group 5 (81°–100°) corresponded to 10 points of the Likert scale.

We considered the pre-lockdown period from 26 February to 10 March 2020 and the lockdown period from 12 March to 3 May 2020 [26].

### 2.5. Statistical Analysis

Univariate differences were tested using Pearson’s Chi square test for categorical variables and nonparametric Wilcoxon Mann–Whitney test for independent continuous variables. We carried out multivariate ordered logistic regression to investigate the association between the socioeconomic characteristics, the variables investigated in the questionnaire and two outcomes (information and behavior change).

We carried out multiple imputations with chained equations [27] to generate values for missing data points such as sex, age, region and attitude to face the pandemic. The percentage of missing data was 25%.

All variables included in the models as predictors of outcomes were used to predict missing values [27,28]. Data were assumed to be “missing at random” [28]. Twenty-five datasets were imputed. Outcomes were not imputed. Data analysis was performed with STATA 13.0 SE (Stata Corporation, College Station, TX, USA).

## 3. Results

**Figure 1:**
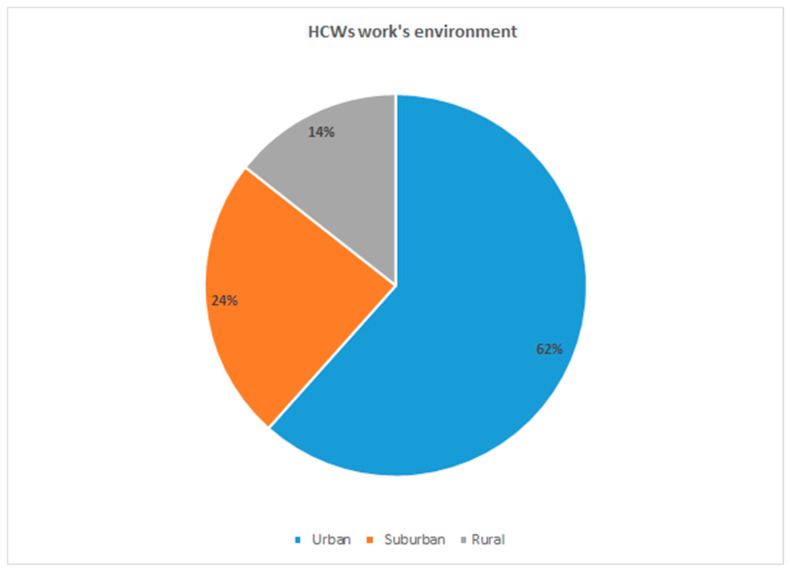
Healthcare workers’ environment of work.

A total of 958 participants were included. Of these, 320 (33.4%) were general practitioners, 248 (25.9%) were pediatricians and 390 (40.7%) were other health professionals. Most responders filled in the questionnaire during the pre-lockdown period (72.4%). The characteristics of the participants are shown in Table 1. Most respondents were from Northern Italy (48.1%), followed by Central Italy (29.9%) and, lastly, Southern Italy (22.0%), and the majority of respondents were female (61.8%). Most of HCWs work in an urban environment (62%) (Figure 1).

**Figure 2:**
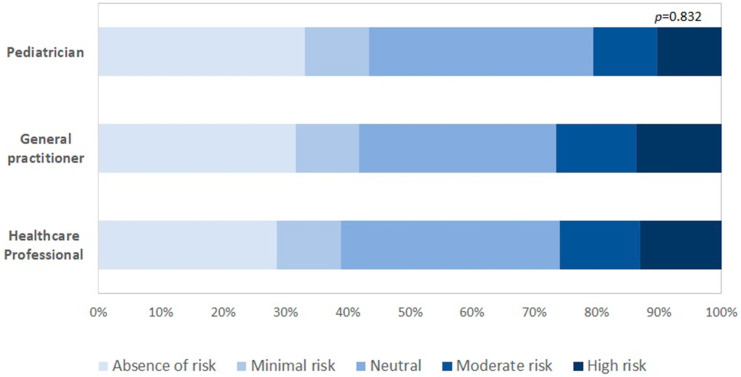
Proportion of HCWs reporting patients afraid of the novel coronavirus pandemic.

Patients were reportedly more afraid of COVID-19 during the lockdown period (15.8%) than in the pre-lockdown period (8.4%) (*p* < 0.001) (Table 2). Health professionals reported a higher level of perceived risk of contracting COVID-19 from their patients than pediatricians (Figure 2).

**Figure 3:**
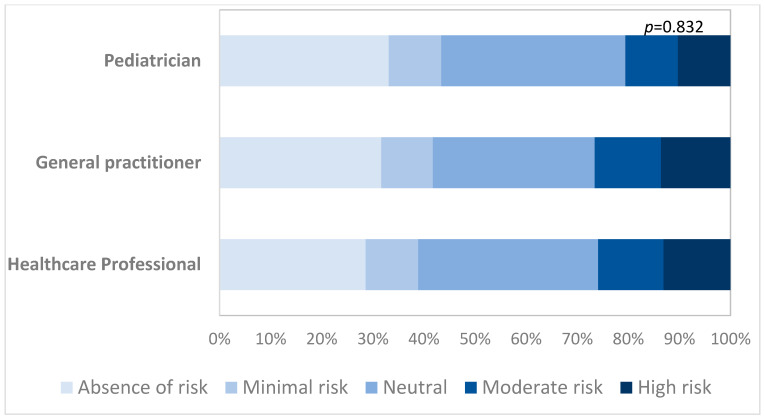
Perception of the risk to visit a COVID-19 patient by type of HCWs.

According to respondents, the risk of having contact with a patient affected by COVID-19 was far higher in the lockdown period than the pre-lockdown period (19.4% vs 10.2%, *p* = 0.002) (Table 2). This risk was higher for general practitioners than for pediatricians and other health professionals, but this difference was not statistically significant (Figure 3).

Respondents reported having changed their clinical practice more in the lockdown period (81.1%) than in pre-lockdown (46.3%) (Table 2). Particularly, they increased the use of masks (87.1%, *p* < 0.001), disinfection and sanitization of doctors’ offices (75.8%, *p* < 0.001), the use of protective glasses and alcoholic hand solution (71.2%, *p* < 0.001) and hand washing (31.8%, *p* = 0.028) (Table 2).

Among participants, pediatricians were those who felt most well informed by health authorities (96.4%).

The multivariate model analyzing information showed that, in the older age groups, information increases with age, particularly in respondents aged over 66 (OR 2.03, *p* = 0.040). Pediatricians are the most well informed (OR 1.78, *p* = 0.015) and institutional e-mails are the most reported method for them to be informed (OR 1.81, *p* < 0.001). Participants who declared not to feel ready to face the COVID-19 emergency also reported less information (OR 0.13, *p* < 0.001) (Table 3).

The multivariate model analyzing behavior change showed that general practitioners changed their behavior less than health professionals (OR 0.54, *p* = 0.008). Participants from Southern Italy changed their behavior less than those from Northern Italy (OR 0.67, *p* = 0.049). Age positively affected behavior change, and respondents ranging from 46 to 55 years (OR = 1.81, *p* = 0.024) of age showed more willingness to change their behavior, as did those aged 56–65 (OR = 1.81, *p* = 0.023) (Table 3).

Respondents declared to have changed their behavior more during the lockdown period than pre-lockdown (OR 6.22, *p* < 0.001). Moreover, those who used the ministry toll-free number to inform themselves, reported the greatest behavior change (OR 2.03, *p* = 0.001).

## 4. Discussion

COVID-19 is a global health problem, especially among HCWs. Italy was the first European country to face COVID-19 pandemic, with considerable differences in terms of organization and management strategies throughout the country, resulting in heterogeneous levels of performance across regional health systems.

Our study shows that HCWs have a sufficient level of information about COVID-19, and participants frequently reported a change in their behavior in clinical practice during the pandemic. Doctors had a higher level of information and, amongst those, pediatricians were better informed.

Healthcare professionals, being in contact with patients, played a crucial role in the transmission of COVID-19; thousands of HCW’s, mainly general practitioners, were affected by COVID-19 and died while caring for COVID-19 positive patients [26]. This could be due to several factors: lack of personal protective equipment (PPE), poor information of the virus containment measures, especially in the first pandemic wave, and heavy workloads. For this reason, we investigated HCWs’ level of information and perceptions of the prevention and control of the COVID-19 pandemic. It is crucial for HCWs to be prepared and to apply all IPC in facing COVID-19 [29,30,31] considering that the prevalence of the infection among HCWs exceeded 10% in Italy [32,33,34] with a consequent loss of capacity for hospitals to respond adequately to the pandemic.

Information and perceptions of COVID-19 varied across different categories of HCWs. Other studies have shown that the majority of HCWs had a good level of information on COVID-19 and showed a positive attitude related to their sense of readiness to confront the disease and implemented good practices towards COVID-19 [30,35,36,37].

Moreover, doctors declared they had significantly modified their clinical practice during the pandemic period compared to other health professionals, showing a higher impact of the pandemic on medical doctors’ daily routines. HCWs needed deeper knowledge, and they tried to obtain this knowledge through national health authorities, other colleagues’ opinions or social networks.

Doctors were also much more confident in the information coming from the Italian National Health Authorities compared to other health professionals. This indicates that the COVID-19-related updates posted by official health authorities had positive implications for improving doctors’ information levels. Obtaining information from institutional sources is crucial for disseminating reliable data about the emerging COVID-19 infection and is essential for HCWs’ preparedness and response [29].

During the pre-lockdown and lockdown periods, all health professionals were informed about the best IPC to be adopted for the containment of COVID-19. Most respondents received their information from institutional channels, while 38.5% had obtained information from other colleagues and 15.8% from social networks.

The findings of this study suggest a significant gap between the amount of information available on COVID-19 and the depth of information among HCWs, particularly regarding disinfection of doctors’ offices and contact surfaces, use of protective glasses and use of alcoholic solution for hand hygiene. All these practices were not extensively applied in the pre-lockdown period because they were not routinely used by HCWs before the pandemic, yet their use significantly increased during lockdown. This could be due to a low penetration of information and trust in the messages of the health authorities at the beginning of the pandemic, which were perceived only later as important and vital to fight the pandemic. However, it is important to note that, as expected, the change in IPC measures was mainly driven by those measures less used in the clinical routine (such as use of protective glasses, and use of alcoholic solution for hand hygiene), while well known IPC measures (such as hand washing) increased less.

Other studies, investigating scope of information level, risk perception and practices, showed that doctors exhibited higher knowledge scores than nurses and paramedics [30]. Information, risk perception and practice regarding the use of masks and their differences were found to be inadequate mostly among medical staff.

HCWs showed a positive attitude but moderate-to-poor level of information and practice regarding the use of face masks [35,38,39].

Two interesting results in our study were that the majority of respondents declared to have radically changed their work habits in the lockdown period; moreover, the use of surgical masks among respondents increased much more in the lockdown period compared to the use of FFP1 or FFP3 masks.

However, this study has some limitations that should be considered. The survey was conducted through the use of an online platform and disseminated through social networks; therefore, the type of sampling used may not be representative of all Italian HCWs and could be biased towards respondents with a positive knowledge, attitude and practice.

## 5. Conclusions

We identified a good level of information among respondent HCWs who felt to be adequately prepared to deal with the pandemic. However, considering that the global threat of COVID-19 continues to emerge and that healthcare professionals are at high risk of COVID-19 transmission to and from patients, greater efforts through educational campaigns that target HCWs are urgently needed.

## Figures and Tables

**Table 1:** Participants’ demographics (*n* = 958).

	Total (*n* = 958)		Pre-Lockdown (*n* = 694)		Lockdown (*n* = 264)		
	n	%	n	%	n	%	*p*
**Type of participants**							0.022
Non medical staff	390	40.7	267	38.5	123	46.6	
Medical staff	568	59.3	427	61.5	141	53.4	
**Profession**							<0.001
Allied healthcare	390	40.7	267	38.5	123	46.6	
General Practitioner	320	33.4	216	31.1	104	39.4	
Pediatrician	248	25.9	211	30.4	37	14.0	
**Age (range)**							0.043
Median (IQR)	52.0 (36–62)		51.0 (35–62)		54.0 (40–62)		
**Age (in years)**							0.041
≤35	215	24.1	170	26.2	45	18.7	
36–45	150	16.8	111	17.1	39	16.2	
46–55	136	15.3	87	13.4	49	20.3	
56–65	312	35.0	227	34.9	85	35.3	
≥66	78	8.8	55	8.5	23	9.5	
**Sex**							<0.001
Male	341	38.2	221	34.0	120	49.6	
Female	551	61.8	429	66.0	122	50.4	
**Area**							0.545
North Italy	427	48.1	318	48.9	109	45.8	
Central Italy	266	29.9	195	30.0	71	29.8	
South Italy	195	22.0	137	21.1	58	24.4	
Organization *							0.828
Hospital	222	57.2	155	58.1	67	55.4	
Residential Care Facility	60	15.5	39	14.6	21	17.4	
Private institution	36	9.3	27	10.1	9	7.4	
Community healthcare center	33	8.5	22	8.2	11	9.1	
Other	37	9.5	24	9.0	13	10.7	

* only for healthcare professionals.

**Table 2:** Information level, risk perception and practice of HCWs by survey study period in Italy, univariate analysis.

	Total (*n* = 958)		Pre-Lockdown (*n* = 694)		Lockdown (*n* = 264)		
	n	%	n	%	n	%	*p*
**Are the patients you come in contact with, scared of the COVID-19 pandemic?**							<0.001
Not at all frightened	237	25.2	197	28.9	40	15.4	
A little scared	274	29.1	219	32.1	55	21.2	
Neutral	221	23.5	149	21.8	72	27.8	
Quite frightened	111	11.8	60	8.8	51	19.7	
Very scared	98	10.4	57	8.4	41	15.8	
**In the last week, which was for you the risk to come in contact with patients affected by COVID-19?**							0.002
Absence of risk	264	31.1	216	33.5	48	23.3	
Minimal risk	87	10.2	69	10.7	18	8.7	
Neutral	290	34.1	219	34.0	71	34.5	
Moderate risk	103	12.1	74	11.5	29	14.1	
High risk	106	12.5	66	10.2	40	19.4	
**What containment measures were put in place in your work place?**							
Isolation of the patient (yes)	72	85.7	29	96.7	43	82.7	0.063
Contact quarantine (yes)	40	47.6	18	60.0	22	40.7	0.090
Administration of nasal swabs for close contacts	35	41.7	15	50.0	20	35.2	0.185
Healthcare worker quarantine (yes)	25	29.8	12	40.0	13	24.1	0.126
Social containment measures (yes)	21	25.0	-	-	21	25.0	<0.001
Use of PPE (yes)	4	4.8	2	6.7	2	3.7	0.143
**Since the start of the COVID-19 pandemic, have you changed the way you work?**							<0.001
Yes, absolutely	519	55.8	313	46.3	206	81.1	
Yes, moderately	335	36.0	291	43.1	44	17.3	
No, not really	51	5.5	49	7.2	2	0.8	
No, not at all	21	2.3	19	2.8	2	0.8	
I don’t know	4	0.4	4	0.6	-	-	
What have you changed in your clinical practice? *							
Increased frequency of handwashing (yes)	256	26.7	172	24.8	84	31.8	0.028
Increased office disinfection (yes)	624	65.1	424	61.1	200	75.8	<0.001
Increased use of masks (yes)	628	65.6	398	57.4	230	87.1	<0.001
Increased use of protective glasses (yes)	440	45.9	252	36.3	188	71.2	<0.001
Increased use of alcohol based hand solution (yes)	467	48.8	279	40.2	188	71.2	<0.001
**Do you believe that the information released by international health authorities regarding the COVID-19 pandemic in China has been clear enough?**							0.100
Yes, absolutely	152	18.0	111	18.2	41	17.7	
Yes, moderately	330	39.2	255	41.7	75	32.3	
No, not really	283	33.6	191	31.3	92	39.7	
No, not at all	71	8.4	49	8.0	22	9.5	
I don’t know	7	0.8	5	0.8	2	0.9	
**Do you believe that the information disseminated by national and regional health authorities regarding the risks associated with COVID-19 for the Italian population have been sufficiently clear?**							0.129
Yes, absolutely	174	20.9	122	20.1	52	23.2	
Yes, moderately	354	42.5	271	44.6	83	37.1	
No, not really	222	26.7	163	26.8	59	26.3	
No, not at all	78	9.4	49	8.1	29	12.9	
I don’t know	4	0.5	3	0.5	1	0.5	
**Do you think that the definition of a suspected case of a COVID-19 infection is sufficiently clear?**							0.750
Yes, absolutely	185	22.4	133	22.1	52	23.4	
Yes, moderately	353	42.8	265	43.9	88	39.6	
No, not really	235	28.5	170	28.2	65	29.3	
No, not at all	48	5.8	32	5.3	16	7.2	
I don’t know	4	0.5	3	0.5	1	0.5	
**Do you think you have been sufficiently informed by the national health authorities on how to behave if you are faced with a suspected COVID-19 case?**							0.477
Yes, absolutely	195	23.3	147	24.3	48	20.9	
Yes, moderately	375	44.9	276	45.5	99	43.0	
No, not really	204	24.4	138	22.8	66	28.7	
No, not at all	59	7.1	43	7.1	16	7.0	
I don’t know	3	0.4	2	0.3	1	0.4	
**Which of the following protective devices is most suitable to avoid the risk of transmission of COVID-19?**							<0.001
Surgical mask	63	7.5	24	3.9	39	16.8	
FFP1 mask	17	2.0	13	2.1	4	1.7	
FFP2 mask	16	1.9	7	1.1	9	3.9	
FFP3 mask	642	75.9	510	83.1	132	56.9	
Gas-masks	1	0.1	1	0.2	-	-	
All the above	107	12.7	59	9.6	48	20.7	
How do you keep yourself informed about operational guidelines? *							
Emails sent by health authorities (yes)	595	62.1	450	64.8	145	54.9	0.005
Proactive search for information on official institutions’ websites (yes)	521	54.4	360	51.9	161	61.0	0.011
Proactive search for information through Ministry of Health toll-free number (yes)	156	16.3	113	16.3	43	16.3	0.998
Emails received from scientific companies (yes)	262	27.4	190	27.4	72	27.3	0.974
Exchange of information with other colleagues (yes)	496	51.8	339	48.8	157	59.9	0.003
Medical-scientific publications (yes)	291	30.4	203	29.2	88	33.3	0.220
Social networks (yes)	151	15.8	116	16.7	35	13.3	0.189
From patients (yes)	7	0.7	4	0.6	3	1.1	0.363
**Which of the following social networks do you find most reliable to follow updates on the COVID-19 pandemic?**							
Facebook (yes)	111	13.2	89	14.6	22	9.6	0.055
LinkedIn (yes)	22	2.6	10	1.6	12	5.2	0.004
Instagram (yes)	16	1.9	12	2.0	4	1.7	0.829
Twitter (yes)	28	3.3	18	2.9	10	4.3	0.315
They are not reliable for updates (yes)	684	81.4	495	81.1	189	82.2	0.733
**Do you think that the measures put in place by the health authorities to contain the spread of the pandemic in Italy are appropriate?**							0.380
Yes, absolutely	211	25.0	149	24.3	62	27.1	
Yes, moderately	395	46.9	284	46.2	111	48.5	
No, not really	181	21.5	141	23.0	40	17.5	
No, not at all	44	5.2	30	4.9	14	6.1	
I don’t know	12	1.4	10	1.6	2	0.9	
**Now that the COVID-19 pandemic has arrived in Italy, do you feel ready to face it?**							0.234
Yes, absolutely	61	7.3	40	6.6	21	9.3	
Yes, moderately	308	37.0	216	35.6	92	40.7	
No, not really	351	42.1	266	43.8	85	37.6	
No, not at all	94	11.3	69	11.4	25	11.1	
I don’t know	19	2.3	16	2.6	3	1.3	
**With reference to the COVID-19 pandemic, do you think that the importance given to, and spread by, the media and society in general is excessive?**							0.001
Yes, absolutely	131	16.0	107	18.0	24	10.6	
Yes, moderately	216	26.3	167	28.1	49	21.7	
No, not really	265	32.3	186	31.3	79	35.0	
No, not at all	199	24.3	126	21.2	73	32.3	
I don’t know	9	1.1	8	1.4	1	0.4	

* more than one answer was possible.

**Table 3:** Odd ratios of information and behavioral change.

	Information			Behavior Change		
	adjOR	*p*-Value	95% CI	adjOR	*p*-Value	95% CI
**Age**						
≤35	Reference			Reference		
36–45	0.89	0.614	0.56–1.42	1.71	0.027 *	1.06–2.76
46–55	1.10	0.701	0.67–1.80	1.81	0.024 *	1.08–3.04
56–65	1.60	0.061	0.98–2.62	1.81	0.023 *	1.09–3.02
≥66	2.03	0.040 *	1.03–4.00	1.49	0.241	0.76–2.93
**Sex**						
Male	Reference			Reference		
Female	1.23	0.225	0.88–1.70	1.26	0.184	0.90–1.78
**Profession**						
Healthcare Professional	Reference			Reference		
General practitioner	1.03	0.886	0.67–1.58	0.54	0.008 *	0.35–0.85
Pediatrician	1.78	0.015 *	1.12–2.85	0.64	0.083	0.39–1.07
**Region**						
North Italy	Reference			Reference		
Central Italy	1.35	0.078	0.97–1.88	0.73	0.066	0.51–1.02
South Italy	1.41	0.076	0.93–2.08	0.67	0.049 *	0.46–1.00
**Period**						
Pre-lockdown	Reference			Reference		
Lockdown	1.16	0.377	0.84–1.59	6.22	<0.001 *	4.22–9.17
**Email sent by health authorities**						
No	Reference			Reference		
Yes	1.81	<0.001 *	1.32–2.50	1.28	0.153	0.91–1.79
**Ministry of Health toll-free number**						
No	Reference			Reference		
Yes	1.21	0.304	0.84–1.73	2.03	0.001 *	1.35–3.06
**Now that the pandemic from COVID-19 has arrived in Italy, do you feel ready to face it?**						
Yes, absolutely	Reference			Reference		
Yes, moderately	0.56	0.064	0.31–1.03	1.88	0.028 *	1.07–3.30
No, not really	0.27	<0.001 *	0.15–0.50	1.76	0.047 *	1.01–3.08
No, not at all	0.13	<0.001 *	0.06–0.26	1.35	0.390	0.68–2.64
I don’t know	0.20	0.003 *	0.07–0.57	1.82	0.288	0.60–5.51

* significant *p*-value. The model was adjusted also for sex, email sent by health authorities, official institutions’ websites, emails received from scientific companies, exchange of information with other colleagues, medical-scientific publications, social network.

## Data Availability

All data generated or analyzed during this study are included in this published.

## Abbreviations

HCW	Healthcare workers
IPC	Infection prevention and control
COVID-19	Coronavirus Disease 2019
PPE	Personal protective equipment
